# 2339. COVID-19 Vaccination in Adults and Children. Cross-Sectional Study of Adults', Pediatricians' and Parents' perceptions and Acceptance of COVID-19 Vaccine in Colombia

**DOI:** 10.1093/ofid/ofad500.1961

**Published:** 2023-11-27

**Authors:** Margarita Cortés, Andrea Puentes, Mónica Arenas, Carlos Arango, José Brea, Claudia Beltran, Roberto Debbag

**Affiliations:** Tecnofarma, Bogota, Cundinamarca, Colombia; Tecnofarma, Bogota, Cundinamarca, Colombia; Tecnofarma, Bogota, Cundinamarca, Colombia; Fundación Salutia, Bogota, Cundinamarca, Colombia; Slipe - Sociedad Latinoamericana De Infectología Pediátrica, Santo Domingo, Distrito Nacional, Dominican Republic; Universidad de Antioquia, Medellin, Antioquia, Colombia; Slipe - Sociedad Latinoamericana De Infectología Pediátrica, Santo Domingo, Distrito Nacional, Dominican Republic

## Abstract

**Background:**

COVID-19 (C-19) immunization started in Colombia in February 2021 for adults; in October 2021 for children over 3 years old (yo), and since January 2023 the recommendation was extended to children 6 m - 3 yo.

At the time of this study, C-19 vaccine (C-19V) coverage rates were still low in Colombia: adults, first booster: 43.7%; children over 3 yo 67.8% first dose, 47.9% second dose.

The objective of this study was to assess C-19V acceptance in Colombia, among pediatricians, parents of children up to 18 yo and adults.

**Methods:**

In January 2023 we carried out 3 online surveys on C-19V among adults, parents and pediatricians in Colombia.

**Results:**

*Adults*

655 adults over 18 yo participated in the study: 80% lived in an urban area, 80% had completed high school, 93% had received C-19V (50% complete primary schedule, 35% first booster); 83% had been contacted by their social security to be reminded about the importance of vaccination. Among the non-vaccinated, 71% did not want to receive C-19V; over 41% said they did not want or did not need it. In the vaccinated group, 66% reported confidence in the vaccines and 55% said they willed would complete their C-19V schedule.

*Pediatricians*

767 pediatricians answered the survey, 52.1% work in private practice. Perception of C-19V risks among pediatricians was low for both adults and children; vaccine acceptance was lower for healthy children than for adults (69.4 vs 96.4%). The main causes for vaccine hesitancy were the lack of scientific information, fear of immediate and long-term side effects, and non-severity of the disease in children (57.3%, 20.8% and 33.3%, and 21.8%, respectively).

*Parents*

Of the 646 parents, 86.2% were mothers, 57.7% had postgraduate degrees, 89.4% had vaccinated their children against C-19. The main cause for vaccine hesitancy was vaccine safety (36.6%); 71.2% were informed about how vaccines work, and 64.8% regarding the risks of C-19 in children.

**Conclusion:**

These results show a high acceptance of C-19V in Colombia. However, coverage rates for were low at the time of the study. This might suggest that besides vaccine confidence, other factors impacting coverage rates should be evaluated, as vaccine access.

**Disclosures:**

**All Authors**: No reported disclosures

